# Evaluation of dosimetric consistency between pre- and postoperative CT-guided noncoplanar template-assisted ¹²⁵I seed implantation in recurrent and metastatic thoracic tumors: a retrospective study

**DOI:** 10.7717/peerj.21324

**Published:** 2026-05-22

**Authors:** Lingyun Qiu, Yucheng Li, Rong Wu, Yinan Chen, Ziguo Zhang, Wenming Zhan, Yinghao Zhang, Qiang Li, Aihong Bi, Limin Luo, Weijun Chen, Zhiwen Yan

**Affiliations:** 1 Cancer Center, Department of Radiation Oncology, Zhejiang Provincial People’s Hospital (Affiliated People’s Hospital), Hangzhou Medical College, Hangzhou, Zhejiang, China; 2 Department of Breast Surgery, Hangzhou TCM Hospital Affiliated to Zhejiang Chinese Medical University, Hangzhou Hospital of Traditional Chinese Medicine, Hangzhou, Zhejiang, China; 3 School of Medical Technology, Yunnan Engineering Vocational College, Yunnan, China; 4 School of National Defence Science & Technology, Southwest University of Science and Technology, Mianyang, Sichuan, China; 5 Cancer Center, Clinical Medical Engineering Department, Zhejiang Provincial People’s Hospital (Affiliated People’s Hospital), Hangzhou Medical College, Hangzhou, Zhejiang, China

**Keywords:** Noncoplanar template, Radioactive seed implantation, Thoracic tumors, Dosimetry, CT-guided brachytherapy, Retrospective study

## Abstract

**Objective:**

Recurrent and metastatic thoracic malignancies remain challenging to manage because of their proximity to vital structures and the impact of respiratory motion on dose accuracy. This retrospective study aimed to assess the dosimetric consistency between preoperative planning and postoperative verification in computed tomography (CT)-guided 3D-printed noncoplanar template (3DPNCT)-assisted ¹²⁵I seed implantation.

**Methods:**

Clinical and dosimetric data from 32 patients who underwent 3DPNCT-assisted seed implantation were retrospectively reviewed. Preoperative treatment plans were compared with postoperative dose verification to evaluate deviations in key indices, including target coverage, dose conformity, and homogeneity. Statistical comparisons were performed using appropriate paired analyses, and agreement between planned and delivered doses was further evaluated through Bland–Altman analysis.

**Results:**

The mean D90 decreased from 142.47 ± 28.90 Gy to 135.06 ± 31.66 Gy (*p* = 0.022). The mean V100 decreased from 93.19 ± 14.74% to 91.48 ± 12.37% (*p* = 0.441), and V150 from 73.81 ± 16.20% to 69.30 ± 17.61% (*p* = 0.059). No significant differences were observed in V200, CI, or EI (all *p* > 0.05). The homogeneity index (HI) increased slightly from 0.222 ± 0.114 to 0.257 ± 0.130 (*p* = 0.049).

**Conclusions:**

CT-guided 3DPNCT-assisted ¹²⁵I seed implantation achieves robust dosimetric reproducibility and favorable plan accuracy in thoracic tumors. Despite minor deviations related to respiratory motion and intraoperative uncertainties, this approach provides a reliable and clinically feasible option for managing complex thoracic malignancies. Further prospective studies incorporating motion management and real-time adaptive planning are warranted to optimize its clinical application.

## Introduction

Thoracic malignancies present formidable challenges in radiation oncology, especially when recurrence or metastasis occurs in previously irradiated regions. Anatomical proximity to critical organs and respiratory-induced motion limit the margin for error in dose delivery (Zhao et al., 2020; Siegel et al., 2025). For patients who are unsuited for additional external beam radiotherapy or surgery intervention, ¹²⁵I seed brachytherapy offers a minimally invasive alternative, enabling steep dose gradients and localized tumoricidal effects while sparing adjacent healthy tissues (Qiu et al., 2021; Chen et al., 2021; Chuang et al., 2024). Continuous low-dose-rate emission from implanted seeds is particularly suitable for lesions in constrained anatomical settings (Chuang et al., 2024).

However, achieving high dosimetric accuracy in the thoracic region remains technically demanding. Respiratory motion, heterogeneous tissue densities, and proximity to organs at risk, such as the heart and large vessels, can lead to deviations in seed placement, resulting in altered dose distributions (Jiang et al., 2018; Wang et al., 2020). Traditional freehand implantation or coplanar templates often lack the flexibility to accommodate three-dimensional anatomical complexity, introducing uncertainties and operator dependence (Yu et al., 2022; Wang et al., 2023).

Three-dimensional printing technology offers a promising solution to these challenges. Patient-specific noncoplanar templates (3DPNCTs), designed from detailed preoperative computed tomography (CT) data, can guide needle trajectories with submillimeter precision. By allowing multiple nonparallel insertion paths, these templates enable customized needle alignment even in constrained anatomical regions (Nuyts et al., 2024; Ji et al., 2024; Mishra et al., 2024; Wang et al., 2025). Clinical applications of 3D-printed templates in brachytherapy have been explored in head and neck, gynecologic, hepatic, and interstitial settings, with reported improvements in dose conformity and reproducibility (Han et al., 2021; Perez-Calatayud et al., 2012; Nath et al., 2016; Arce et al., 2025). Compared with conventional coplanar templates or freehand needle insertion, individualized noncoplanar templates provide predefined needle trajectories that can be accurately aligned with the patient surface. This guidance reduces operator dependence and improves reproducibility of needle placement, particularly in anatomically complex thoracic regions where freehand insertion may be limited by ribs, vessels, or critical organs. Nonetheless, most existing studies focus on feasibility or applications in nonthoracic sites, leaving a knowledge gap in evaluating dosimetric consistency specifically in thoracic tumor applications.

Although several prior works have examined dosimetric verification for ¹²⁵I seed implantation assisted by 3D-printed templates—including comparisons of pre- and postoperative plans—systematic evaluations in thoracic settings are limited (Zhang et al., 2024; Qiu et al., 2025; Li et al., 2025). The influence of respiratory motion, intraoperative positional shifts, and tissue deformation on final dose delivery remains underexplored in this anatomical domain. Thus, objective evidence of reproducibility is essential before wider clinical adoption. Here, we retrospectively evaluated the dosimetric concordance between preoperative planning and postoperative verification in patients who underwent CT-guided 3DPNCT-assisted ¹²⁵I seed implantation for recurrent and metastatic thoracic tumors. By comparing indices of conformity, coverage, and homogeneity and analyzing deviation patterns, we aim to assess the reliability of this technique and to identify potential contributors to residual discrepancies.

## Materials and Methods

### Patient selection

We performed a retrospective review of patients who underwent CT-guided 3DPNCT-assisted ¹²⁵I seed implantation for recurrent or metastatic thoracic tumors at our institution between January 2018 and December 2024. Eligible patients were adults (≥18 years) with histologically confirmed thoracic malignancies who had previously received external beam radiotherapy and who presented with localized recurrent or metastatic lesions. The exclusion criteria included uncorrected bleeding disorders, a Karnofsky performance status <60, incomplete dosimetric data, or uncontrolled systemic disease in Table 1. Demographic, clinical, and treatment data—including tumor location, prior EBRT dose, prescription dose, and imaging metrics—were extracted from institutional records.

**Table 1:** General characteristics of the 32 cases included in this review.

Characteristic	Value
Gender	
Male	21
Female	11
Age	63 (31–81)
KPS score	80 (70–90)
Tumor stage	
II	11
III	14
IV	7
Prescribed dose (Gy)	145 Gy (120–160 Gy)
Seed activity	0.7 (0.5–0.8)
Tumor diameter (cm)	3.4 (1.8–6.2)

**Note:**

Continuous variables are presented as median (range); categorical variables as *n* (%).

### Implantation procedure

**Figure 1:**
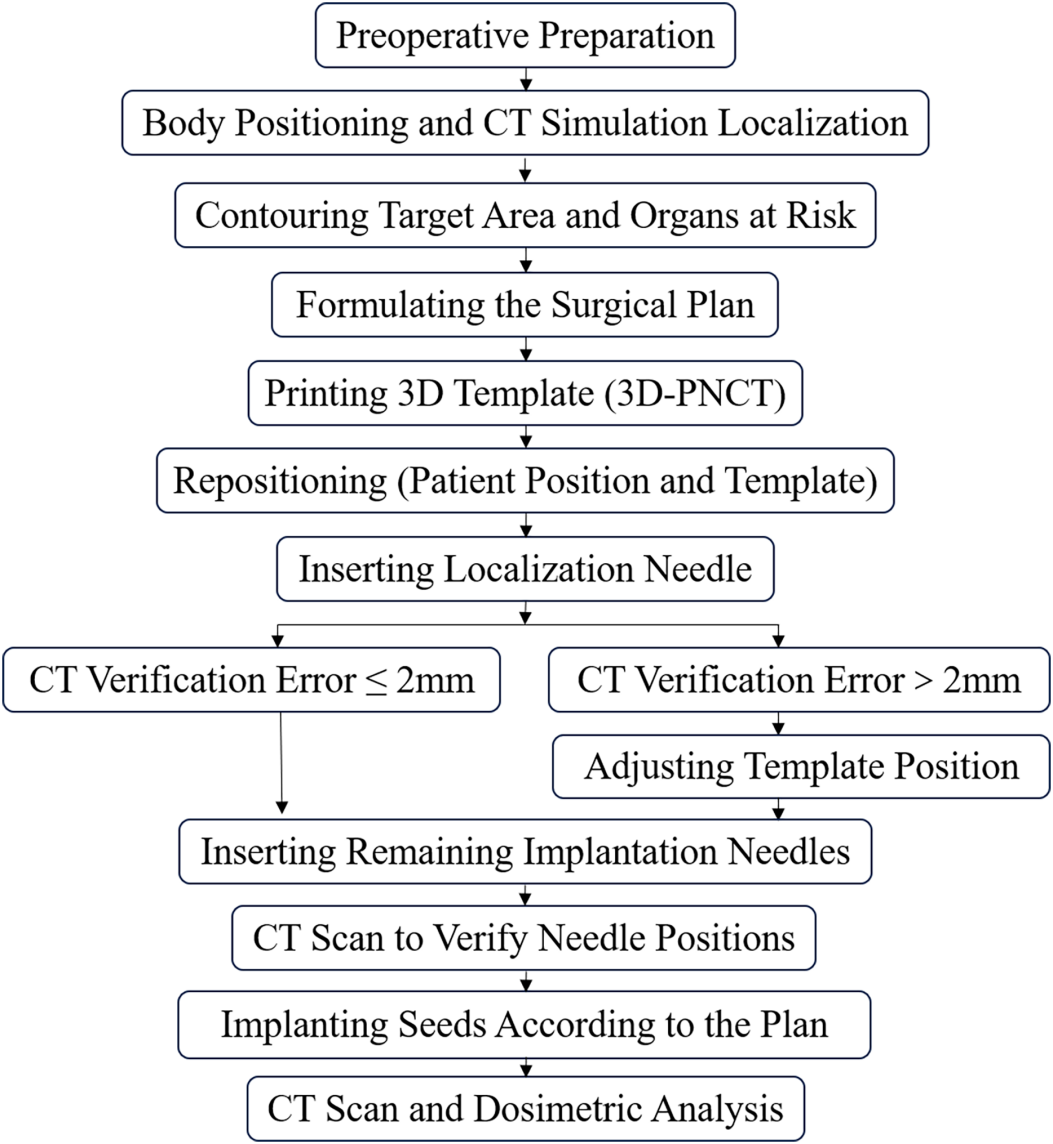
Implant technical processes.

**Figure 2:**
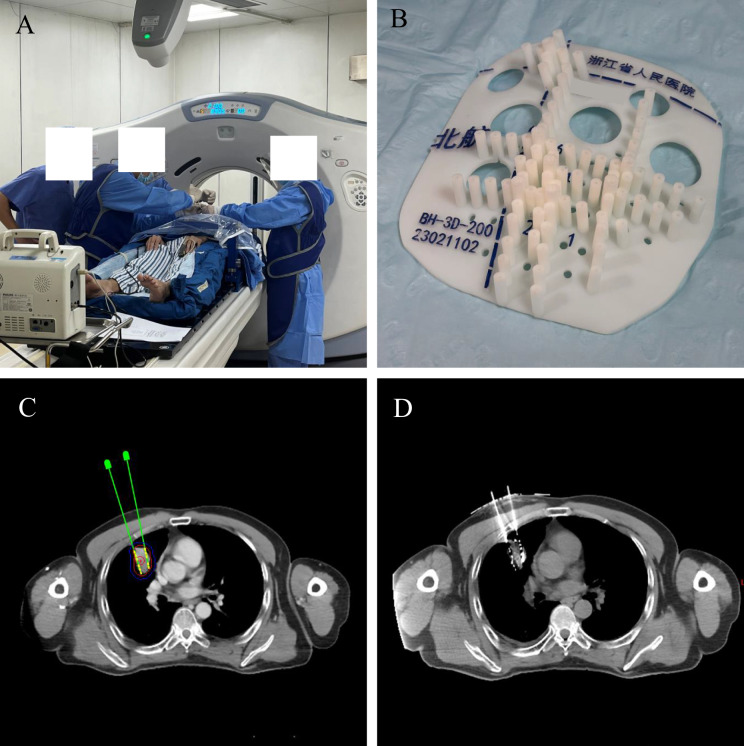
(A–D) The clinical operation of 3D-PNCT assisted CT-guided RISI.

All implantations were performed by experienced radiation oncologists and medical physicists (Fig. 1). Patients were immobilized with a vacuum cushion and underwent CT simulation (slice thickness 3–5 mm; General Electric Company, American). CT images were acquired with a slice thickness of 3–5 mm during end-expiration breath-hold when possible to minimize respiratory motion. Patients unable to perform breath-holding underwent CT scanning during quiet breathing with respiratory motion monitoring. The gross tumor volume (GTV) and organs at risk (OARs) were delineated in the treatment planning system (TPS; Beijing Astro Technology Ltd., Beijing, China). GTV was delineated on CT images based on visible tumor boundaries. OARs were considered close to the target when located within approximately 1 cm of the GTV or along the planned needle trajectory. No additional planning target volume margin was added because the implantation procedure directly targeted the visible lesion. Noncoplanar needle trajectories were devised to optimize target coverage while minimizing OAR exposure, with attention to anticipated respiratory motion. Patient-specific templates were fabricated using stereolithographic 3D printing. The templates incorporated auxiliary registration channels at 5-mm intervals to enhance surface alignment and intraoperative adjustments. After sterilization, the template was aligned to surface landmarks and secured; a verification CT scan confirmed alignment within ±2 mm prior to seed placement. Under CT guidance, ¹²⁵I seeds (seeds with an activity ranging from 0.5 to 0.8 mCi; 0.8 × 4.5 mm; Beijing Atom High Tech Co., Ltd., Beijing, China) were implanted along the planned trajectories (Fig. 2). Intraoperative CT was performed as needed to confirm the needle position when necessary to confirm needle position, and minor adjustments were made when deviations exceeded tolerance. Immediately after implantation, CT was performed for seed localization and postoperative dose calculation (van der Pol et al., 2024).

### Dosimetric evaluation and consistency assessment

The prescribed dose (120–160 Gy) was individualized based on tumor size, anatomical location, prior radiotherapy exposure, and treatment intent (*e.g*., salvage or boost therapy). Organs at risk (OARs) included the heart, esophagus, spinal cord, trachea, and major thoracic vessels when located near the target lesion. Image registration was performed primarily based on tumor anatomy and surrounding structures. Seed positions were subsequently verified on postoperative CT images. Postoperative CT images were coregistered to preoperative images using rigid and deformable registration within the TPS. Postimplant CT verification was performed within 1 h after seed implantation to evaluate the delivered dose distribution. Dose–volume histogram (DVH) parameters—including D90; V100; V150; and indices of conformity (CI), external exposure (EI), and homogeneity (HI)—were extracted for both plans. Qualitative comparisons were used to describe the magnitude and direction of deviations. Bland–Altman plots were generated to visualize the agreement ranges and bias trends. Where appropriate, descriptive associations with procedural factors (*e.g*., needle count and lesion site) were explored (Ödén et al., 2024). Paired t-tests were used to compare pre- and postoperative parameters. Agreement between measurements was further evaluated using Bland–Altman analysis.

This retrospective analysis received approval from the Ethics Committee of Zhejiang Provincial People’s Hospital (2024-09-09, No. QT2024223). All study procedures adhered to the ethical standards outlined in the Declaration of Helsinki. Owing to the retrospective design and the anonymization of patient data, the requirement for individual informed consent was waived by the committee. Prior to analysis, all CT datasets were fully deidentified to ensure confidentiality. Data acquisition and processing were conducted between June and August 2025.

## Results

### Patient and treatment characteristics

A total of 32 patients with recurrent or metastatic thoracic malignancies (including 18 lung, eight mediastinal, and six chest wall lesions) were included between January 2018 and December 2024.

The median tumor diameter was 3.4 cm (range 1.8–6.2 cm), and the median prescribed dose was 145 Gy (range 120–160 Gy). All patients underwent successful implantation, and the total number of implanted ¹²⁵I seeds per patient ranged from 32 to 96 (median 54). No serious intraoperative complications, such as pneumothorax >20% or major bleeding, were reported. Minor subcutaneous hematomas occurred in two patients (6.3%) and resolved without intervention.

### Dosimetric concordance between pre- and postoperative plans

**Figure 3:**
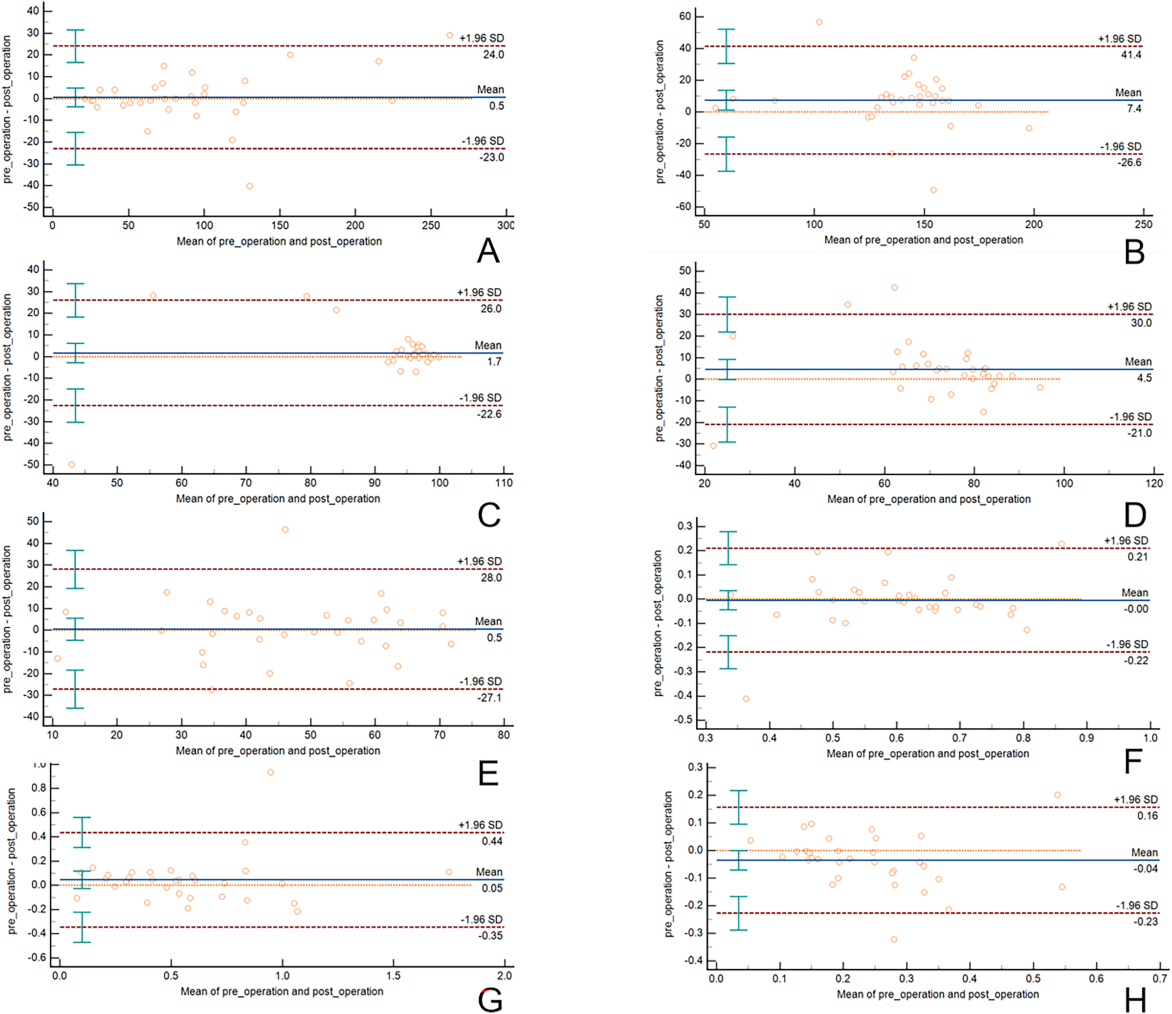
Bland-Altman plot of differences in preoperative planning and postoperative dose parameters. (A) D90, (B) D100, (C) V100, (D) V150, (E) V200, (F) CI, (G) EI, (H) HI.

Table 2 summarizes the pre- and postoperative dosimetric indices. The mean D90 was 142.47 ± 28.9 Gy preoperatively and 135.06 ± 31.66 Gy postoperatively, indicating a mean relative difference of −2.1% (*p* = 0.022). The mean V100 was 93.19 ± 14.74% preoperatively and 91.48 ± 12.37% after implantation (Δ = −0.9%). The V150 decreased slightly from 73.81 ± 16.2% to 69.3 ± 17.61% (Δ = −2.1%, *p* > 0.05), suggesting a mild reduction in hotspot volume. With respect to the quality indices, the conformity index (CI) remained stable (0.6 ± 0.146 *vs*. 0.604 ± 0.127), whereas the homogeneity index (HI) improved marginally (0.222 ± 0.114 *vs*. 0.257 ± 0.13). The external index (EI), which represents the volume of normal tissue receiving ≥ prescription dose, remained low (0.591 ± 0.375 *vs*. 0.545 ± 0.356). Overall, deviations in all dosimetric parameters between the pre- and postoperative plans were within 3%, indicating high procedural reproducibility (Fig. 3).

**Table 2:** Comparison of preoperative and postoperative validation parameters in 32 patients.

Parameter	Preoperative (Mean ± SD)	Postoperative (Mean ± SD)	*P*
Number of seeds	92.66 ± 60.05	92.10 ± 56.39	0.795
D_90_/Gy	142.47 ± 28.90	135.06 ± 31.66	0.022
V_100_/%	93.19 ± 14.74	91.48 ± 12.37	0.441
V_150_/%	73.81 ± 16.20	69.30 ± 17.61	0.059
V_200_/%	46.93 ± 17.60	46.47 ± 17.22	0.854
CI	0.600 ± 0.146	0.604 ± 0.127	0.845
EI	0.591 ± 0.375	0.545 ± 0.356	0.204
HI	0.222 ± 0.114	0.257 ± 0.130	0.049

### Spatial and geometric accuracy

Postimplant CT verification revealed good agreement between the planned and actual seed positions. The mean spatial deviation of the seed centroids was 1.6 ± 0.7 mm, with 93% of the seeds within 2 mm of the planned location. Bland–Altman analysis demonstrated narrow 95% limits of agreement for D90 (−7.4 to +9.1 Gy) and V100 (−2.5% to +3.2%), confirming high consistency. Lesions located near the diaphragm may experience greater motion amplitude due to respiratory excursion, which could contribute to increased geometric uncertainty.

### Dosimetric correlation with tumor site

Subgroup analysis indicated that compared with peripheral lung lesions (mean 1.4 mm), mediastinal lesions exhibited slightly greater geometric deviation (mean 2.1 mm), likely due to respiratory motion affecting all thoracic lesions, with additional influence from cardiac pulsation in mediastinal tumors. Among the included cases, four lesions were located adjacent to the diaphragm. Needle trajectories were adjusted to avoid diaphragmatic penetration, and no procedure-related complications occurred. However, these variations did not translate into clinically relevant dosimetric degradation, as D90 and CI remained within acceptable clinical tolerances across all groups.

## Discussion

This retrospective study assessed the dosimetric consistency between planned and delivered ¹²⁵I seed implantation using CT-guided 3DPNCT in patients with thoracic malignancies. Our findings demonstrate robust dosimetric reproducibility, with close agreement between preoperative and postoperative plans and only minor deviations in hotspot indices. These observations extend prior evidence from head and neck, pelvic, and hepatic applications to the thoracic domain, where respiratory motion and complex organ geometry introduce unique uncertainties (Han et al., 2021; Zhang et al., 2024). The study population included lesions in the lung, mediastinum, and chest wall. Although these locations differ anatomically, subgroup analysis demonstrated comparable dosimetric consistency across sites. In clinical brachytherapy practice, deviations within ±10 Gy for D90 and ±5% for V100 are generally considered acceptable.

Clinical and technical implications: Compared with freehand or coplanar techniques, template guidance provides individualized control over needle paths, reducing operator dependence and improving dose conformity. The tendency toward reduced high-dose hotspots postoperatively suggests that intraoperative adjustments and noncoplanar trajectories can smooth dose gradients, potentially enhancing safety near critical thoracic structures (Ji et al., 2024). The reproducibility observed here underscores the feasibility of integrating 3DPNCTs into thoracic brachytherapy workflows. Respiratory motion remains an important source of uncertainty in thoracic brachytherapy. Although breath-hold CT acquisition was used when possible, residual motion may still contribute to minor geometric deviations. A few outlier points observed in the Bland–Altman plots may be associated with lesions located near highly mobile structures such as the mediastinum or diaphragm.

Comparison with the previous literature: Prior studies reported low deviation rates and high plan reproducibility when individualized 3D-printed templates were used across multiple disease sites. For example, Qiu et al. (2021) and Chen et al. (2021) described consistent conformity indices and limited pre–post differences in template-assisted implants (Mishra et al., 2024). Han et al. (2021) validated lung applications of polylactic templates with high spatial fidelity. Our results are concordant with these observations while emphasizing thoracic-specific considerations such as respiratory motion and soft-tissue deformation (Jiang et al., 2018; Li et al., 2025).

Potential sources of deviation: Residual discrepancies likely arise from respiratory motion during implantation, tissue deformation along needle paths, and minor intraoperative positional shifts. Surface-based template registration may not fully compensate for internal organ motion. Future adoption of respiratory gating, fluoroscopic verification, and deformable image registration could further reduce uncertainties (Cozzarini et al., 2022; Ödén et al., 2024).

Limitations and future work: This single-center retrospective analysis with a limited sample size may have limited generalizability. Static postoperative CT verification did not capture dynamic motion effects; interoperator variability was not quantified. Future prospective studies incorporating respiratory motion management and improved image guidance are warranted (Chua, Leong & Lim, 2010; Zhao et al., 2020; Chen et al., 2021).

## Conclusions

CT-guided 3D-printed noncoplanar template–assisted ¹²⁵I seed implantation demonstrates strong dosimetric reproducibility in recurrent and metastatic thoracic tumors. High concordance between preoperative and postoperative plans supports the clinical feasibility of this approach. Minor deviations associated with respiratory motion and intraoperative uncertainties highlight the value of motion management and adaptive workflows to further optimize outcomes.

## Supplemental Information

10.7717/peerj.21324/supp-1 Supplemental Information 1 Data. The preoperative and postoperative dosimetric parameters for all radioactive seed implantation procedures.

## Additional Information and Declarations

### Data Availability

The following information was supplied regarding data availability:

The original measurement data are available in the Supplemental File.
